# Bis(dimethyl­formamide-κ*O*){4,4′,6,6′-tetra­chloro-2,2-[butane-1,4-di­yl(nitrilo­methanylyl­idene)]diphenolato-κ^4^ *O*,*N*,*N*′,*O*′}nickel(II)

**DOI:** 10.1107/S1600536812028681

**Published:** 2012-06-30

**Authors:** Hadi Kargar, Reza Kia, Amir Adabi Ardakani, Muhammad Nawaz Tahir

**Affiliations:** a Department of Chemistry, Payame Noor University, PO Box 19395-3697 Tehran, I. R. of IRAN; b Department of Chemistry, Science and Research Branch, Islamic Azad University, Tehran, Iran; c Department of Physics, University of Sargodha, Punjab, Pakistan

## Abstract

In the title Schiff base complex, [Ni(C_18_H_14_Cl_4_N_2_O_2_)(C_3_H_7_NO)_2_], the geometry around the Ni^II^ atom is distorted octa­hedral. It is coordinated by the N_2_O_2_ donor atoms of the tetra­dentate Schiff base ligand and the O atoms of two dimethyl­formamide mol­ecules, which are *cis* to one another. The benzene rings are almost normal to each other [dihedral angle = 88.60 (14)°]. The various intra­molecular C—H⋯O hydrogen bonds make *S*(5) and *S*(6) ring motifs. In the crystal, mol­ecules are linked by pairs of weak C—H⋯Cl inter­actions, forming inversion dimers.

## Related literature

For standard bond lengths, see: Allen *et al.* (1987 ▶). For hydrogen-bond motifs, see: Bernstein *et al.* (1995 ▶). For background to Schiff base ligands and their complexes, see: Kargar, Kia, Abbasian *et al.* (2012 ▶); Kargar *et al.* (2011 ▶); Kia *et al.* (2010 ▶). For the crystal structure of the ligand, see: Kargar, Kia, Ardakani *et al.* (2012 ▶).
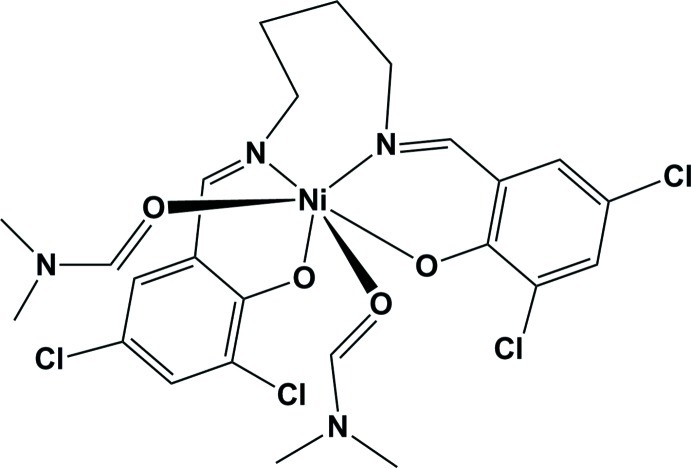


## Experimental

### 

#### Crystal data

[Ni(C_18_H_14_Cl_4_N_2_O_2_)(C_3_H_7_NO)_2_]
*M* *_r_* = 637.01
Monoclinic, 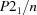

*a* = 9.7392 (11) Å
*b* = 19.165 (2) Å
*c* = 15.0197 (14) Å
β = 93.236 (3)°
*V* = 2799.0 (5) Å^3^
*Z* = 4
Mo *K*α radiation
μ = 1.11 mm^−1^
*T* = 291 K
0.36 × 0.28 × 0.26 mm

#### Data collection

Bruker SMART APEXII CCD area-detector diffractometer
Absorption correction: multi-scan (*SADABS*; Bruker, 2005 ▶) *T*_min_ = 0.690, *T*_max_ = 0.761
23789 measured reflections
6633 independent reflections
4349 reflections with *I* > 2σ(*I*)
*R*_int_ = 0.053

#### Refinement

*R*[*F* ^2^ > 2σ(*F* ^2^)] = 0.044
*wR*(*F* ^2^) = 0.123
*S* = 1.02
6633 reflections
338 parameters
H-atom parameters constrained
Δρ_max_ = 0.39 e Å^−3^
Δρ_min_ = −0.58 e Å^−3^

### 

Data collection: *APEX2* (Bruker, 2005 ▶); cell refinement: *SAINT* (Bruker, 2005 ▶); data reduction: *SAINT*; program(s) used to solve structure: *SHELXS97* (Sheldrick, 2008 ▶); program(s) used to refine structure: *SHELXL97* (Sheldrick, 2008 ▶); molecular graphics: *SHELXTL* (Sheldrick, 2008 ▶); software used to prepare material for publication: *SHELXTL* and *PLATON* (Spek, 2009 ▶).

## Supplementary Material

Crystal structure: contains datablock(s) global, I. DOI: 10.1107/S1600536812028681/su2463sup1.cif

Structure factors: contains datablock(s) I. DOI: 10.1107/S1600536812028681/su2463Isup2.hkl

Additional supplementary materials:  crystallographic information; 3D view; checkCIF report

## Figures and Tables

**Table 1:** Hydrogen-bond geometry (Å, °)

*D*—H⋯*A*	*D*—H	H⋯*A*	*D*⋯*A*	*D*—H⋯*A*
C9—H9*B*⋯O4	0.97	2.58	3.327 (4)	134
C11—H11*B*⋯O4	0.97	2.40	3.057 (4)	125
C19—H19⋯O1	0.93	2.25	2.865 (4)	123
C8—H8*A*⋯Cl3^i^	0.97	2.86	3.753 (3)	153

Symmetry code: (i) 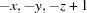.

**Fig. 1.**
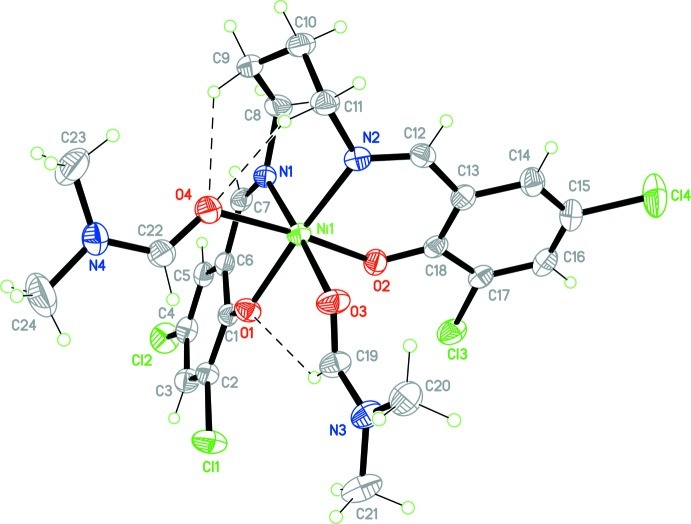
The molecular structure of the title compound, showing 40% probability displacement ellipsoids and the atomic numbering. Dashed lines show the intramolecular C-H···O interactions (see Table 1 for details).

**Fig. 2.**
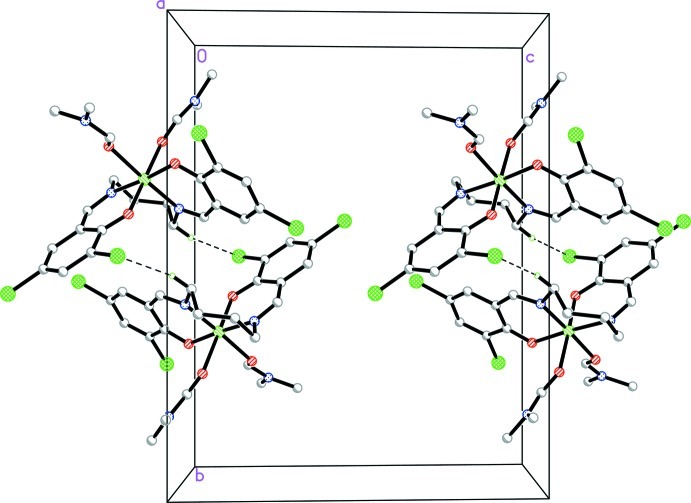
The crystal packing of the title compound viewed along the a axis, showing linking of molecules through weak C—H···Cl interactions (dashed lines; see Table 1 for details) into individual inversion dimers. Only the H atoms involved in these interactions are shown .

## supplementary crystallographic information

### Comment

In continuation of our work on the synthesis and crystal structure analysis of Schiff base ligands and their complexes (Kargar, Kia, Abbasian *et al.*, 2012; Kargar, Kia, Ardakani *et al.*, 2012; Kargar *et al.*, 2011; Kia *et al.*, 2010), we report herein on the synthesize and crystal structure of the title compound.

The asymmetric unit of the title compound, Fig. 1, comprises a Ni^II^ Schiff base complex. The geometry around Ni^II^ is distorted octahedral being coordinated by N_2_O_2_ donor atoms of the tetradentate ligand, 6,6'-((butane-1,4-diylbis(azanylylidene))bis(methanylylidene)) bis(2,4-dichlorophenol) [Kargar, Kia, Ardakani *et al.*, 2012] and by two oxygen atoms of dimethylformamide molecules that are *cis* to one another. The bond lengths (Allen *et al.*, 1987) and angles are within the normal range. The intramolecular C—H···O hydrogen bonds makes *S(5)* and *S(6)* ring motif (Table 1; Bernstein *et al.*, 1995). The substituted benzene rings [C1–C6 and C13–C18] are almost normal [88.60 (14)°] to each other.

In the crystal structure molecules are linked by pairs of weak C—H···Cl interactions into individual inversion dimers (Table 1 and Fig. 2).

### Experimental

The title compound was synthesized by adding 3,5-dichlorosalicylaldehyde-1,4-butylenediimine (1 mmol) to a solution of NiCl_2_ .6H_2_O (1.1 mmol) in ethanol (30 ml). The mixture was refluxed with stirring for 30 min. The resultant solution was filtered. Green prismatic single crystals of the title compound, suitable for *X*-ray structure determination, were obtained by recrystallization from ethanol on slow evaporation of the solvents at room temperature over several days.

### Refinement

The H-atoms were included in calculated positions and treated as riding atoms: C-H = 0.93, 0.96 and 0.97 Å for CH, CH_3_ and CH_2_ H-atoms, respectively, with U_iso_(H) = k × U_eq_(parent C-atom), where k = 1.5 for CH_3_ H-atoms and = 1.2 for other H-atoms.

### Crystal data

**Table:**

[Ni(C_18_H_14_Cl_4_N_2_O_2_)(C_3_H_7_NO)_2_]	*F*(000) = 1312
*M**_r_* = 637.01	*D*_x_ = 1.512 Mg m^−^^3^
Monoclinic, *P*2_1_/*n*	Mo *K*α radiation, λ = 0.71073 Å
Hall symbol: -P 2yn	Cell parameters from 3422 reflections
*a* = 9.7392 (11) Å	θ = 2.8–27.5°
*b* = 19.165 (2) Å	µ = 1.11 mm^−^^1^
*c* = 15.0197 (14) Å	*T* = 291 K
β = 93.236 (3)°	Prism, green
*V* = 2799.0 (5) Å^3^	0.36 × 0.28 × 0.26 mm
*Z* = 4	

### Data collection

**Table:**

Bruker SMART APEXII CCD area-detector diffractometer	6633 independent reflections
Radiation source: fine-focus sealed tube	4349 reflections with *I* > 2σ(*I*)
Graphite monochromator	*R*_int_ = 0.053
φ and ω scans	θ_max_ = 27.9°, θ_min_ = 1.7°
Absorption correction: multi-scan (*SADABS*; Bruker, 2005)	*h* = −12→12
*T*_min_ = 0.690, *T*_max_ = 0.761	*k* = −25→25
23789 measured reflections	*l* = −18→19

### Refinement

**Table:**

Refinement on *F*^2^	Primary atom site location: structure-invariant direct methods
Least-squares matrix: full	Secondary atom site location: difference Fourier map
*R*[*F*^2^ > 2σ(*F*^2^)] = 0.044	Hydrogen site location: inferred from neighbouring sites
*wR*(*F*^2^) = 0.123	H-atom parameters constrained
*S* = 1.02	*w* = 1/[σ^2^(*F*_o_^2^) + (0.0542*P*)^2^ + 0.7005*P*] where *P* = (*F*_o_^2^ + 2*F*_c_^2^)/3
6633 reflections	(Δ/σ)_max_ = 0.001
338 parameters	Δρ_max_ = 0.39 e Å^−^^3^
0 restraints	Δρ_min_ = −0.58 e Å^−^^3^

### Special details

**Table:**

Geometry. All esds (except the esd in the dihedral angle between two l.s. planes) are estimated using the full covariance matrix. The cell esds are taken into account individually in the estimation of esds in distances, angles and torsion angles; correlations between esds in cell parameters are only used when they are defined by crystal symmetry. An approximate (isotropic) treatment of cell esds is used for estimating esds involving l.s. planes.
Refinement. Refinement of F^2^ against ALL reflections. The weighted R-factor wR and goodness of fit S are based on F^2^, conventional R-factors R are based on F, with F set to zero for negative F^2^. The threshold expression of F^2^ > 2sigma(F^2^) is used only for calculating R-factors(gt) etc. and is not relevant to the choice of reflections for refinement. R-factors based on F^2^ are statistically about twice as large as those based on F, and R- factors based on ALL data will be even larger.

### Fractional atomic coordinates and isotropic or equivalent isotropic displacement parameters (Å^2^)

**Table:**

	*x*	*y*	*z*	*U*_iso_*/*U*_eq_	
Ni1	0.03675 (3)	0.167782 (17)	0.39862 (2)	0.03835 (12)	
Cl1	0.42524 (9)	0.25307 (4)	0.57798 (6)	0.0652 (2)	
Cl2	0.34269 (10)	0.05880 (5)	0.83006 (6)	0.0714 (3)	
Cl3	0.40497 (8)	0.00038 (5)	0.36167 (6)	0.0626 (2)	
Cl4	0.18116 (14)	−0.08010 (6)	0.04365 (7)	0.0929 (4)	
O1	0.1739 (2)	0.19835 (9)	0.49423 (13)	0.0440 (5)	
O2	0.1643 (2)	0.09054 (10)	0.36689 (14)	0.0477 (5)	
O3	0.1363 (2)	0.23648 (11)	0.31056 (15)	0.0545 (5)	
O4	−0.0881 (2)	0.25534 (10)	0.43674 (16)	0.0536 (5)	
N1	−0.0578 (2)	0.10534 (11)	0.48708 (16)	0.0393 (5)	
N2	−0.0946 (2)	0.14157 (11)	0.29273 (16)	0.0413 (5)	
N3	0.3381 (3)	0.28212 (13)	0.27093 (18)	0.0524 (6)	
N4	−0.1082 (3)	0.34747 (13)	0.5284 (2)	0.0599 (7)	
C1	0.2073 (3)	0.16609 (13)	0.56753 (19)	0.0375 (6)	
C2	0.3275 (3)	0.18564 (14)	0.6196 (2)	0.0438 (7)	
C3	0.3696 (3)	0.15504 (15)	0.6988 (2)	0.0507 (7)	
H3	0.4488	0.1700	0.7307	0.061*	
C4	0.2906 (3)	0.10097 (15)	0.7302 (2)	0.0477 (7)	
C5	0.1725 (3)	0.08039 (14)	0.68435 (19)	0.0440 (7)	
H5	0.1200	0.0449	0.7074	0.053*	
C6	0.1289 (3)	0.11139 (13)	0.60378 (19)	0.0395 (6)	
C7	0.0006 (3)	0.08645 (13)	0.5616 (2)	0.0425 (7)	
H7	−0.0451	0.0524	0.5927	0.051*	
C8	−0.1887 (3)	0.07074 (15)	0.4602 (2)	0.0499 (7)	
H8A	−0.2166	0.0421	0.5093	0.060*	
H8B	−0.1741	0.0400	0.4103	0.060*	
C9	−0.3051 (3)	0.12120 (16)	0.4337 (2)	0.0539 (8)	
H9A	−0.3881	0.1052	0.4601	0.065*	
H9B	−0.2824	0.1666	0.4589	0.065*	
C10	−0.3356 (3)	0.12976 (16)	0.3339 (2)	0.0535 (8)	
H10A	−0.3411	0.0837	0.3071	0.064*	
H10B	−0.4254	0.1513	0.3245	0.064*	
C11	−0.2327 (3)	0.17269 (15)	0.2847 (2)	0.0485 (7)	
H11A	−0.2630	0.1758	0.2222	0.058*	
H11B	−0.2290	0.2196	0.3090	0.058*	
C12	−0.0633 (3)	0.10065 (14)	0.22963 (19)	0.0416 (6)	
H12	−0.1270	0.0977	0.1813	0.050*	
C13	0.0590 (3)	0.05857 (14)	0.22437 (19)	0.0424 (7)	
C14	0.0659 (3)	0.01658 (16)	0.1478 (2)	0.0510 (7)	
H14	−0.0042	0.0189	0.1032	0.061*	
C15	0.1742 (4)	−0.02746 (16)	0.1383 (2)	0.0556 (8)	
C16	0.2789 (3)	−0.03246 (15)	0.2039 (2)	0.0540 (8)	
H16	0.3522	−0.0628	0.1972	0.065*	
C17	0.2736 (3)	0.00781 (14)	0.2790 (2)	0.0447 (7)	
C18	0.1638 (3)	0.05554 (14)	0.2943 (2)	0.0411 (6)	
C19	0.2586 (3)	0.24959 (15)	0.3267 (2)	0.0493 (7)	
H19	0.2982	0.2358	0.3817	0.059*	
C20	0.2846 (4)	0.30430 (19)	0.1833 (2)	0.0675 (10)	
H20A	0.1949	0.2845	0.1712	0.101*	
H20B	0.3450	0.2887	0.1391	0.101*	
H20C	0.2783	0.3543	0.1818	0.101*	
C21	0.4789 (4)	0.3010 (2)	0.2978 (3)	0.0774 (11)	
H21A	0.5021	0.2828	0.3563	0.116*	
H21B	0.4875	0.3509	0.2987	0.116*	
H21C	0.5400	0.2818	0.2562	0.116*	
C22	−0.0370 (3)	0.30254 (15)	0.4834 (2)	0.0518 (8)	
H22	0.0583	0.3067	0.4872	0.062*	
C23	−0.2567 (4)	0.3464 (2)	0.5240 (3)	0.0882 (14)	
H23A	−0.2892	0.3089	0.4859	0.132*	
H23B	−0.2912	0.3899	0.5003	0.132*	
H23C	−0.2885	0.3396	0.5827	0.132*	
C24	−0.0397 (5)	0.4028 (2)	0.5811 (4)	0.1100 (18)	
H24A	−0.0736	0.4474	0.5606	0.165*	
H24B	0.0577	0.4005	0.5746	0.165*	
H24C	−0.0583	0.3970	0.6428	0.165*	

### Atomic displacement parameters (Å^2^)

**Table:**

	*U*^11^	*U*^22^	*U*^33^	*U*^12^	*U*^13^	*U*^23^
Ni1	0.03646 (19)	0.03847 (19)	0.0406 (2)	−0.00509 (14)	0.00601 (15)	0.00000 (15)
Cl1	0.0587 (5)	0.0703 (5)	0.0656 (6)	−0.0261 (4)	−0.0058 (4)	0.0115 (4)
Cl2	0.0795 (6)	0.0831 (6)	0.0507 (5)	0.0078 (5)	−0.0038 (4)	0.0170 (4)
Cl3	0.0420 (4)	0.0766 (6)	0.0693 (6)	0.0060 (4)	0.0048 (4)	0.0036 (4)
Cl4	0.1229 (9)	0.0960 (8)	0.0603 (6)	0.0309 (7)	0.0097 (6)	−0.0273 (5)
O1	0.0462 (11)	0.0416 (10)	0.0439 (12)	−0.0094 (8)	0.0011 (9)	0.0038 (9)
O2	0.0433 (11)	0.0566 (12)	0.0432 (12)	0.0064 (9)	0.0019 (9)	−0.0071 (10)
O3	0.0435 (12)	0.0663 (13)	0.0537 (14)	−0.0126 (10)	0.0046 (10)	0.0147 (11)
O4	0.0486 (12)	0.0433 (11)	0.0691 (15)	−0.0007 (9)	0.0056 (11)	−0.0119 (10)
N1	0.0391 (12)	0.0368 (11)	0.0426 (15)	−0.0064 (9)	0.0079 (10)	−0.0031 (10)
N2	0.0363 (12)	0.0409 (12)	0.0466 (15)	−0.0010 (9)	0.0024 (10)	0.0035 (11)
N3	0.0482 (15)	0.0560 (15)	0.0540 (17)	−0.0036 (12)	0.0129 (12)	0.0131 (12)
N4	0.0649 (18)	0.0504 (15)	0.0648 (19)	0.0059 (13)	0.0066 (15)	−0.0137 (13)
C1	0.0394 (14)	0.0321 (12)	0.0416 (17)	0.0031 (11)	0.0080 (12)	−0.0032 (12)
C2	0.0434 (16)	0.0412 (14)	0.0471 (19)	−0.0034 (12)	0.0054 (13)	−0.0020 (13)
C3	0.0451 (16)	0.0573 (18)	0.049 (2)	0.0048 (14)	−0.0011 (14)	−0.0009 (15)
C4	0.0537 (18)	0.0508 (16)	0.0386 (18)	0.0111 (14)	0.0041 (14)	0.0046 (13)
C5	0.0542 (18)	0.0398 (14)	0.0393 (17)	0.0007 (12)	0.0138 (14)	−0.0016 (12)
C6	0.0453 (15)	0.0350 (13)	0.0394 (17)	0.0007 (11)	0.0123 (12)	−0.0021 (11)
C7	0.0463 (16)	0.0362 (14)	0.0468 (19)	−0.0054 (12)	0.0190 (14)	0.0010 (12)
C8	0.0482 (17)	0.0455 (16)	0.056 (2)	−0.0161 (13)	0.0061 (14)	0.0006 (14)
C9	0.0392 (16)	0.0607 (19)	0.063 (2)	−0.0134 (14)	0.0131 (14)	−0.0137 (15)
C10	0.0354 (15)	0.0563 (18)	0.069 (2)	−0.0025 (13)	0.0039 (14)	−0.0054 (16)
C11	0.0392 (15)	0.0496 (16)	0.056 (2)	0.0017 (12)	−0.0025 (14)	−0.0013 (14)
C12	0.0405 (15)	0.0442 (15)	0.0398 (17)	−0.0041 (12)	−0.0010 (12)	0.0014 (12)
C13	0.0447 (16)	0.0435 (15)	0.0396 (17)	−0.0009 (12)	0.0069 (13)	0.0062 (12)
C14	0.0596 (19)	0.0535 (18)	0.0401 (18)	−0.0003 (14)	0.0052 (14)	0.0011 (14)
C15	0.070 (2)	0.0538 (18)	0.0440 (19)	0.0072 (16)	0.0124 (17)	−0.0045 (15)
C16	0.0560 (19)	0.0482 (16)	0.060 (2)	0.0041 (14)	0.0239 (17)	0.0032 (15)
C17	0.0400 (15)	0.0465 (15)	0.0486 (18)	−0.0013 (12)	0.0109 (13)	0.0057 (13)
C18	0.0384 (14)	0.0417 (14)	0.0443 (18)	−0.0061 (11)	0.0125 (12)	0.0060 (13)
C19	0.0445 (17)	0.0552 (17)	0.0483 (19)	−0.0039 (13)	0.0056 (14)	0.0167 (14)
C20	0.075 (2)	0.075 (2)	0.054 (2)	−0.0091 (19)	0.0167 (19)	0.0089 (18)
C21	0.049 (2)	0.090 (3)	0.095 (3)	−0.0093 (19)	0.020 (2)	0.023 (2)
C22	0.0494 (18)	0.0416 (15)	0.065 (2)	−0.0016 (13)	0.0079 (15)	−0.0036 (15)
C23	0.070 (3)	0.073 (3)	0.126 (4)	0.011 (2)	0.042 (3)	−0.005 (2)
C24	0.119 (4)	0.080 (3)	0.129 (5)	0.011 (3)	−0.007 (3)	−0.056 (3)

### Geometric parameters (Å, º)

**Table:**

Ni1—O1	1.993 (2)	C8—H8A	0.9700
Ni1—O2	2.0069 (19)	C8—H8B	0.9700
Ni1—N1	2.046 (2)	C9—C10	1.520 (5)
Ni1—N2	2.046 (2)	C9—H9A	0.9700
Ni1—O3	2.1370 (19)	C9—H9B	0.9700
Ni1—O4	2.1684 (19)	C10—C11	1.520 (4)
Cl1—C2	1.742 (3)	C10—H10A	0.9700
Cl2—C4	1.753 (3)	C10—H10B	0.9700
Cl3—C17	1.738 (3)	C11—H11A	0.9700
Cl4—C15	1.748 (3)	C11—H11B	0.9700
O1—C1	1.288 (3)	C12—C13	1.444 (4)
O2—C18	1.279 (3)	C12—H12	0.9300
O3—C19	1.229 (3)	C13—C14	1.408 (4)
O4—C22	1.232 (4)	C13—C18	1.425 (4)
N1—C7	1.279 (4)	C14—C15	1.364 (4)
N1—C8	1.473 (3)	C14—H14	0.9300
N2—C12	1.280 (4)	C15—C16	1.381 (5)
N2—C11	1.470 (3)	C16—C17	1.370 (4)
N3—C19	1.328 (4)	C16—H16	0.9300
N3—C20	1.451 (4)	C17—C18	1.436 (4)
N3—C21	1.452 (4)	C19—H19	0.9300
N4—C22	1.315 (4)	C20—H20A	0.9600
N4—C23	1.445 (5)	C20—H20B	0.9600
N4—C24	1.462 (5)	C20—H20C	0.9600
C1—C2	1.421 (4)	C21—H21A	0.9600
C1—C6	1.423 (4)	C21—H21B	0.9600
C2—C3	1.368 (4)	C21—H21C	0.9600
C3—C4	1.389 (4)	C22—H22	0.9300
C3—H3	0.9300	C23—H23A	0.9600
C4—C5	1.365 (4)	C23—H23B	0.9600
C5—C6	1.393 (4)	C23—H23C	0.9600
C5—H5	0.9300	C24—H24A	0.9600
C6—C7	1.450 (4)	C24—H24B	0.9600
C7—H7	0.9300	C24—H24C	0.9600
C8—C9	1.526 (4)		
			
O1—Ni1—O2	89.39 (8)	C11—C10—C9	116.1 (3)
O1—Ni1—N1	90.68 (9)	C11—C10—H10A	108.3
O2—Ni1—N1	91.73 (9)	C9—C10—H10A	108.3
O1—Ni1—N2	174.92 (8)	C11—C10—H10B	108.3
O2—Ni1—N2	90.14 (9)	C9—C10—H10B	108.3
N1—Ni1—N2	94.39 (9)	H10A—C10—H10B	107.4
O1—Ni1—O3	87.48 (8)	N2—C11—C10	111.5 (2)
O2—Ni1—O3	89.95 (8)	N2—C11—H11A	109.3
N1—Ni1—O3	177.50 (9)	C10—C11—H11A	109.3
N2—Ni1—O3	87.46 (9)	N2—C11—H11B	109.3
O1—Ni1—O4	86.86 (8)	C10—C11—H11B	109.3
O2—Ni1—O4	175.86 (8)	H11A—C11—H11B	108.0
N1—Ni1—O4	90.08 (8)	N2—C12—C13	127.9 (3)
N2—Ni1—O4	93.44 (9)	N2—C12—H12	116.1
O3—Ni1—O4	88.13 (8)	C13—C12—H12	116.1
C1—O1—Ni1	126.97 (17)	C14—C13—C18	120.9 (3)
C18—O2—Ni1	128.06 (19)	C14—C13—C12	116.2 (3)
C19—O3—Ni1	118.2 (2)	C18—C13—C12	122.8 (3)
C22—O4—Ni1	120.2 (2)	C15—C14—C13	120.8 (3)
C7—N1—C8	116.6 (2)	C15—C14—H14	119.6
C7—N1—Ni1	122.67 (18)	C13—C14—H14	119.6
C8—N1—Ni1	120.01 (19)	C14—C15—C16	120.8 (3)
C12—N2—C11	116.3 (3)	C14—C15—Cl4	120.5 (3)
C12—N2—Ni1	124.1 (2)	C16—C15—Cl4	118.6 (2)
C11—N2—Ni1	119.52 (19)	C17—C16—C15	119.2 (3)
C19—N3—C20	121.1 (3)	C17—C16—H16	120.4
C19—N3—C21	121.1 (3)	C15—C16—H16	120.4
C20—N3—C21	117.7 (3)	C16—C17—C18	123.7 (3)
C22—N4—C23	121.5 (3)	C16—C17—Cl3	118.8 (2)
C22—N4—C24	121.1 (3)	C18—C17—Cl3	117.5 (2)
C23—N4—C24	117.4 (3)	O2—C18—C13	125.1 (3)
O1—C1—C2	120.3 (2)	O2—C18—C17	120.3 (3)
O1—C1—C6	124.4 (3)	C13—C18—C17	114.6 (3)
C2—C1—C6	115.3 (3)	O3—C19—N3	124.4 (3)
C3—C2—C1	124.2 (3)	O3—C19—H19	117.8
C3—C2—Cl1	119.2 (2)	N3—C19—H19	117.8
C1—C2—Cl1	116.6 (2)	N3—C20—H20A	109.5
C2—C3—C4	118.1 (3)	N3—C20—H20B	109.5
C2—C3—H3	121.0	H20A—C20—H20B	109.5
C4—C3—H3	121.0	N3—C20—H20C	109.5
C5—C4—C3	120.7 (3)	H20A—C20—H20C	109.5
C5—C4—Cl2	119.6 (2)	H20B—C20—H20C	109.5
C3—C4—Cl2	119.7 (3)	N3—C21—H21A	109.5
C4—C5—C6	121.5 (3)	N3—C21—H21B	109.5
C4—C5—H5	119.3	H21A—C21—H21B	109.5
C6—C5—H5	119.3	N3—C21—H21C	109.5
C5—C6—C1	120.2 (3)	H21A—C21—H21C	109.5
C5—C6—C7	116.9 (2)	H21B—C21—H21C	109.5
C1—C6—C7	122.9 (3)	O4—C22—N4	124.4 (3)
N1—C7—C6	128.2 (2)	O4—C22—H22	117.8
N1—C7—H7	115.9	N4—C22—H22	117.8
C6—C7—H7	115.9	N4—C23—H23A	109.5
N1—C8—C9	113.9 (2)	N4—C23—H23B	109.5
N1—C8—H8A	108.8	H23A—C23—H23B	109.5
C9—C8—H8A	108.8	N4—C23—H23C	109.5
N1—C8—H8B	108.8	H23A—C23—H23C	109.5
C9—C8—H8B	108.8	H23B—C23—H23C	109.5
H8A—C8—H8B	107.7	N4—C24—H24A	109.5
C10—C9—C8	115.3 (3)	N4—C24—H24B	109.5
C10—C9—H9A	108.4	H24A—C24—H24B	109.5
C8—C9—H9A	108.4	N4—C24—H24C	109.5
C10—C9—H9B	108.4	H24A—C24—H24C	109.5
C8—C9—H9B	108.4	H24B—C24—H24C	109.5
H9A—C9—H9B	107.5		
			
O2—Ni1—O1—C1	−69.9 (2)	C4—C5—C6—C7	−178.8 (2)
N1—Ni1—O1—C1	21.8 (2)	O1—C1—C6—C5	−179.0 (2)
O3—Ni1—O1—C1	−159.9 (2)	C2—C1—C6—C5	−0.8 (4)
O4—Ni1—O1—C1	111.9 (2)	O1—C1—C6—C7	−0.6 (4)
O1—Ni1—O2—C18	−161.0 (2)	C2—C1—C6—C7	177.6 (2)
N1—Ni1—O2—C18	108.4 (2)	C8—N1—C7—C6	179.8 (3)
N2—Ni1—O2—C18	14.0 (2)	Ni1—N1—C7—C6	9.7 (4)
O3—Ni1—O2—C18	−73.5 (2)	C5—C6—C7—N1	−177.9 (3)
O1—Ni1—O3—C19	27.0 (2)	C1—C6—C7—N1	3.7 (4)
O2—Ni1—O3—C19	−62.4 (2)	C7—N1—C8—C9	129.0 (3)
N2—Ni1—O3—C19	−152.6 (2)	Ni1—N1—C8—C9	−60.6 (3)
O4—Ni1—O3—C19	113.9 (2)	N1—C8—C9—C10	101.6 (3)
O1—Ni1—O4—C22	15.5 (2)	C8—C9—C10—C11	−74.4 (3)
N1—Ni1—O4—C22	106.2 (2)	C12—N2—C11—C10	93.9 (3)
N2—Ni1—O4—C22	−159.4 (2)	Ni1—N2—C11—C10	−88.3 (3)
O3—Ni1—O4—C22	−72.0 (2)	C9—C10—C11—N2	60.4 (4)
O1—Ni1—N1—C7	−18.0 (2)	C11—N2—C12—C13	−173.9 (3)
O2—Ni1—N1—C7	71.5 (2)	Ni1—N2—C12—C13	8.4 (4)
N2—Ni1—N1—C7	161.7 (2)	N2—C12—C13—C14	178.4 (3)
O4—Ni1—N1—C7	−104.8 (2)	N2—C12—C13—C18	2.3 (4)
O1—Ni1—N1—C8	172.2 (2)	C18—C13—C14—C15	−0.8 (4)
O2—Ni1—N1—C8	−98.3 (2)	C12—C13—C14—C15	−177.0 (3)
N2—Ni1—N1—C8	−8.1 (2)	C13—C14—C15—C16	0.6 (5)
O4—Ni1—N1—C8	85.4 (2)	C13—C14—C15—Cl4	179.3 (2)
N1—Ni1—N2—C12	−105.1 (2)	C14—C15—C16—C17	−0.4 (5)
O3—Ni1—N2—C12	76.6 (2)	Cl4—C15—C16—C17	−179.1 (2)
O4—Ni1—N2—C12	164.6 (2)	C15—C16—C17—C18	0.3 (4)
O2—Ni1—N2—C11	169.0 (2)	C15—C16—C17—Cl3	179.3 (2)
N1—Ni1—N2—C11	77.3 (2)	Ni1—O2—C18—C13	−8.8 (4)
O3—Ni1—N2—C11	−101.0 (2)	Ni1—O2—C18—C17	172.30 (18)
O4—Ni1—N2—C11	−13.1 (2)	C14—C13—C18—O2	−178.4 (3)
Ni1—O1—C1—C2	165.65 (18)	C12—C13—C18—O2	−2.4 (4)
Ni1—O1—C1—C6	−16.2 (4)	C14—C13—C18—C17	0.6 (4)
O1—C1—C2—C3	179.0 (3)	C12—C13—C18—C17	176.6 (2)
C6—C1—C2—C3	0.6 (4)	C16—C17—C18—O2	178.6 (3)
O1—C1—C2—Cl1	−1.0 (3)	Cl3—C17—C18—O2	−0.4 (3)
C6—C1—C2—Cl1	−179.33 (19)	C16—C17—C18—C13	−0.4 (4)
C1—C2—C3—C4	0.6 (4)	Cl3—C17—C18—C13	−179.41 (19)
Cl1—C2—C3—C4	−179.4 (2)	Ni1—O3—C19—N3	169.0 (2)
C2—C3—C4—C5	−1.7 (4)	C20—N3—C19—O3	−1.0 (5)
C2—C3—C4—Cl2	178.5 (2)	C21—N3—C19—O3	174.7 (3)
C3—C4—C5—C6	1.6 (4)	Ni1—O4—C22—N4	−161.8 (3)
Cl2—C4—C5—C6	−178.6 (2)	C23—N4—C22—O4	−2.2 (5)
C4—C5—C6—C1	−0.3 (4)	C24—N4—C22—O4	−178.9 (4)

### Hydrogen-bond geometry (Å, º)

**Table:**

*D*—H···*A*	*D*—H	H···*A*	*D*···*A*	*D*—H···*A*
C9—H9*B*···O4	0.97	2.58	3.327 (4)	134
C11—H11*B*···O4	0.97	2.40	3.057 (4)	125
C19—H19···O1	0.93	2.25	2.865 (4)	123
C8—H8*A*···Cl3^i^	0.97	2.86	3.753 (3)	153

Symmetry code: (i) −*x*, −*y*, −*z*+1.
